# Breaking Barriers in Cranioplasty: 3D Printing in Low and Middle-Income Settings—Insights from Zenica, Bosnia and Herzegovina

**DOI:** 10.3390/medicina59101732

**Published:** 2023-09-27

**Authors:** Hakija Bečulić, Denis Spahić, Emir Begagić, Ragib Pugonja, Rasim Skomorac, Aldin Jusić, Edin Selimović, Anes Mašović, Mirza Pojskić

**Affiliations:** 1 Department of Neurosurgery, Cantonal Hospital Zenica, 72000 Zenica, Bosnia and Herzegovina; 2 Department of Anatomy, School of Medicine, University of Zenica, 72000 Zenica, Bosnia and Herzegovina; dr-skomorac@kbze.ba (R.S.); masovicanes@gmail.com (A.M.); 3 Department of Constructions and CAD Technologies, School of Mechanical Engineering, University of Zenica, 72000 Zenica, Bosnia and Herzegovina; denis.spahic@size.ba; 4 iDEAlab, School of Mechanical Engineering, University of Zenica, 72000 Zenica, Bosnia and Herzegovina; 5 Deparment of General Medicine, School of Medicine, University of Zenica, 72000 Zenica, Bosnia and Herzegovina; rpugonja@gmail.com; 6 Department of Surgery, School of Medicine, University of Zenica, 72000 Zenica, Bosnia and Herzegovina; edin.selimovic@unze.ba; 7 Department of Neurosurgery, University Hospital Marburg, Baldinger Str., 35033 Marburg, Germany

**Keywords:** cranioplasty, 3D, print, low-income, middle-income

## Abstract

*Background and Objectives:* Cranial defects pose significant challenges in low and middle-income countries (LIMCs), necessitating innovative and cost-effective craniofacial reconstruction strategies. The purpose of this study was to present the Bosnia and Herzegovina model, showcasing the potential of a multidisciplinary team and 3D-based technologies, particularly PMMA implants, to address cranial defects in a resource-limited setting. *Materials and Methods:* An observational, non-experimental prospective investigation involved three cases of cranioplasty at the Department of Neurosurgery, Cantonal Hospital Zenica, Bosnia and Herzegovina, between 2019 and 2023. The technical process included 3D imaging and modeling with MIMICS software (version 10.01), 3D printing of the prototype, mold construction and intraoperative modification for precise implant fitting. *Results:* The Bosnia and Herzegovina model demonstrated successful outcomes in cranioplasty, with PMMA implants proving cost-effective and efficient in addressing cranial defects. Intraoperative modification contributed to reduced costs and potential complications, while the multidisciplinary approach and 3D-based technologies facilitated accurate reconstruction. *Conclusions:* The Bosnia and Herzegovina model showcases a cost-effective and efficient approach for craniofacial reconstruction in LIMICs. Collaborative efforts, 3D-based technologies, and PMMA implants contribute to successful outcomes. Further research is needed to validate sustained benefits and enhance craniofacial reconstruction strategies in resource-constrained settings.

## 1. Introduction

Cranioplasty (CP) has a rich historical background, with significant advancements dating back to 1668 when van Meekeren successfully utilized a canine cranium for bone grafting [1,2]. Over the years, various materials have been explored, including gold plates, autografts, allografts, and xenografts. Autografts gained prominence with Walther’s introduction in 1821, followed by successful implementations by Macewen and Burrell [1,2,3]. However, challenges persisted with allografts and xenografts due to complications, while metallic substitutes like aluminum, gold, and platinum posed their limitations. The breakthrough came with the introduction of titanium by Simpson in 1965, and subsequently, molded titanium implants and acrylic materials became favored choices for CP [3,4,5].

Decompressive craniectomy (DC) often precedes CP surgery and is commonly employed in cases of traumatic brain injury, intracranial hemorrhage, cerebral infarction, brain edema, and skull fractures [6,7,8]. Extensive clinical case studies and systematic reviews have consistently reported positive outcomes following CP, showcasing the restoration of cranial symmetry, favorable cosmetic results, and notable improvements in neurophysiological function for numerous patients [9,10,11]. Presently, 3D printing plays a pivotal role in generating patient-specific prefabricated cranial implants, resulting in enhanced cosmetic outcomes [12,13]. Nonetheless, despite the progress in image processing and 3D printing, the overall clinical procedure remains time-consuming and costly [14].

The aim of this study is to assess the efficacy and practicality of implementing 3D printing technology as a cost-effective approach to CP in LIMC settings. By evaluating the feasibility, outcomes, and cost-efficiency of 3D-printed patient-specific cranial implants, this research aims to contribute valuable insights that can potentially revolutionize CP practices in resource-limited regions. This study will serve neurosurgeons, medical professionals, and policymakers working in LIMC settings where access to conventional CP methods may be limited due to financial constraints. The findings and recommendations from this research will also be valuable for healthcare organizations, government bodies, and non-governmental organizations involved in improving neurosurgical care and promoting equitable access to advanced medical technologies in resource-constrained areas.

## 2. Materials and Methods

This study was conducted as an observational, non-experimental prospective investigation. It involved three cases of CP performed at the Department of Neurosurgery, Canton Hospital Zenica, Bosnia and Herzegovina, between 2019 and 2023. These three cases represent the first experience with CP in Bosnia and Herzegovina, carried out under limited resources and conditions. The patient treatment process included a multidisciplinary team comprising three neurosurgeons and an engineer. The technical preparation of the implant was executed by the engineer, with continuous consultation with the neurosurgeons.

### 2.1. Patient Evaluation

Prior to the surgical procedure, patients underwent a comprehensive neurological assessment conducted by a neurosurgeon, followed by an evaluative Computed Tomography scan (CT; Somatom Definition AS system from Siemens, Erlangen, Germany). Defect measurements were taken in two diameters: maximal axial and maximal sagittal (mm). Subsequently, the defect area was calculated and classified according to Poukens et al. [15]. This classification is contingent upon three variables: defect size, midline crossing, and orbital rim involvement. The grades are delineated as follows: Grade I for defects not crossing the midline and measuring smaller than 5 cm^2^ without orbital involvement, Grade II for defects not crossing the midline and measuring larger than 5 cm^2^ but smaller or equal to 100 cm^2^ without orbital involvement, Grade III for defects not crossing the midline and measuring larger than 100 cm^2^ without orbital involvement, Grade IV for defects smaller than 5 cm^2^ with orbital involvement, Grade V for defects larger than 5 cm^2^ but smaller or equal to 100 cm^2^ with orbital involvement, and Grade VI for defects larger than 100 cm^2^ with orbital involvement. In addition to classification, an Aesthetic Satisfaction Scoring Scale (ANA) was employed for aesthetic evaluation [16].

### 2.2. Technical Procedure

**Figure 1:**
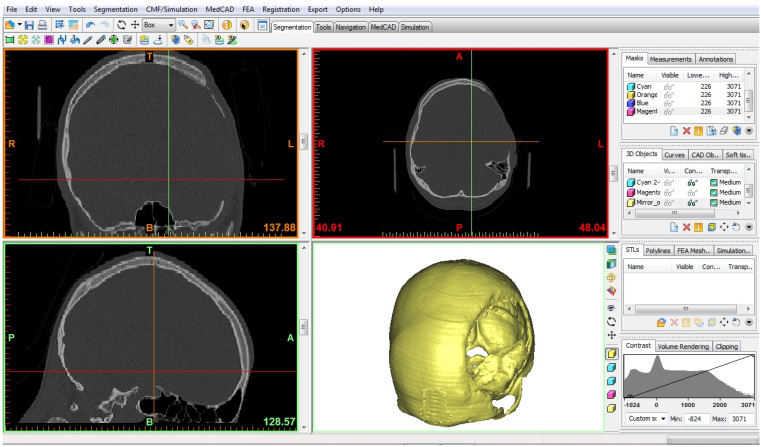
MIMICS software interface.

**Figure 2:**
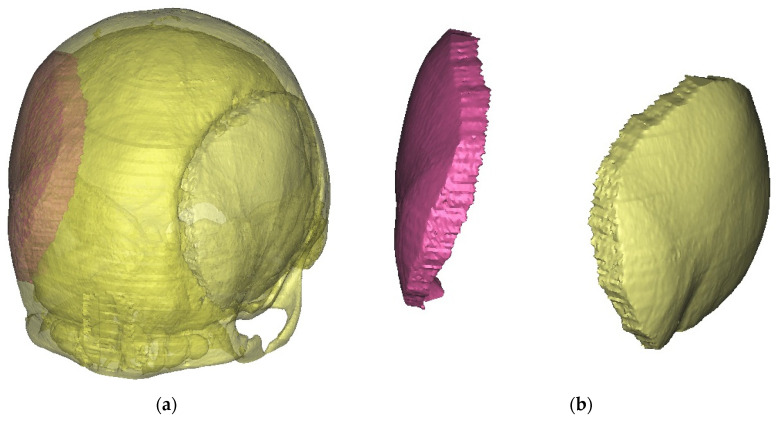
Mirror technique in the preparation of the missing part of the calvaria: (**a**) cutting and checking the match with the defect, and (**b**) isolated reconstructed part corresponding to the defect.

**Figure 3:**
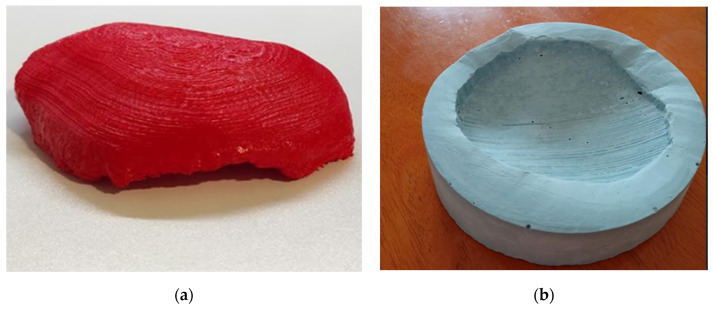
A PLA prototype matching the bone defect (**a**) and a negative prototype—a mold used for intraoperative adaptation of the implant (**b**).

The resulting CT scan files were exported in the Digital Imaging and Communications in Medicine (DICOM) format. Subsequently, the DICOM data were imported into MIMICS software (version 10.01, Materialise’s Interactive Medical Image Control System, Materialise NV, Leuven, Belgium), a specialized tool for interacting with DICOM files. In this study, MIMICS was utilized for image visualization and segmentation, enabling the creation of three-dimensional (3D) renderings of the relevant anatomical structures (Figure 1). By employing cutting and mirroring techniques on the model derived from the unaffected side of the skull, a 3D representation of the missing part of the skull was constructed, effectively closing the defect on the opposite side (Figure 2a,b). The digital 3D model of the absent cranial portion was then exported in STL (Standard Triangle Language) format, suitable for 3D printing purposes (Figure 3a,b).

The 3D prototype of the cranial prosthesis was printed using Polylactic Acid (PLA) plastic on an Ultimaker 2+ 3D printer from Ultimaker, located in Utrecht, The Netherlands (Figure 3a). The 3D-modeling technique was based on Fused Filament Fabrication (FFF) (Video S1). Since the printed implant model was not directly applicable for medical purposes, a negative mold was created using dental plaster (Figure 3b). Dental plaster was chosen due to its sterilization capabilities. This negative mold was then utilized intraoperatively to fabricate an implant that precisely matched the geometry of the cranial defect. The intraoperative prosthesis was made from Polymethyl Methacrylate (PMMA), ensuring the desired fit and functionality during the surgical procedure.

### 2.3. Surgical Procedure

The surgical procedure commenced with an incision on the scalp at the affected site, followed by exploration of the cranial region harboring the defect. Prior to the incision, the operative site underwent disinfection using a cationic detergent, followed by the application of an iodine-based solution. Subsequently, the implant modeling process, which utilized a two-component polymethyl methacrylate (PMMA) mixture, necessitated an extended duration of the surgical intervention. Manual techniques were employed to intricately adapt the PMMA mixture to the cranial defect, ensuring its comprehensive closure.

### 2.4. Follow-Up Assesment

After the surgical treatment, patients were kept under observation in the neurosurgery department at the Canton Hospital Zenica, under the care of the neurosurgical team. The follow-up included wound inspection, neurological examination, and imaging, depending on the available modality, along with aesthetic evaluation. Following discharge, scheduled follow-up appointments were planned with the neurosurgeon at one month, three months post-surgery, and subsequently every six months. Aesthetic Satisfaction Scoring Scale (ANA) was utilized for aesthetic evaluation, with assessments conducted preoperatively, after one month, and after three months [16].

## 3. Results

### 3.1. Case Presentations

**Figure 4:**
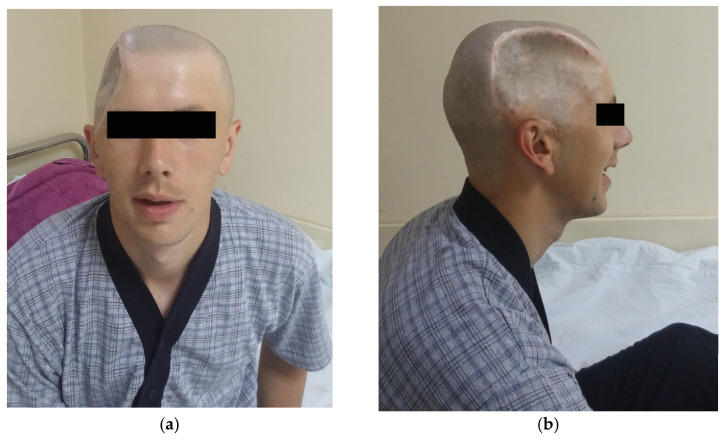
First case with (**a**) frontal and (**b**) lateral aspect preoperative images.

In the first case, a 24-year-old male patient sustained a traumatic brain injury (TBI) following a fall from a height of 4–5 m. One year before the CP surgery, the patient underwent surgery for epidural empyema at the same medical facility, but pathogen isolation was not achieved. The cranial defect involved the frontal, parietal, and temporal bones. The time interval between the injury and CP was 12 months, and the surgical procedure lasted 140 min (Table 1). Additionally, Figure 4a depicts the defect in the frontal aspect, while Figure 4b shows the defect in the lateral aspect.

**Figure 5:**
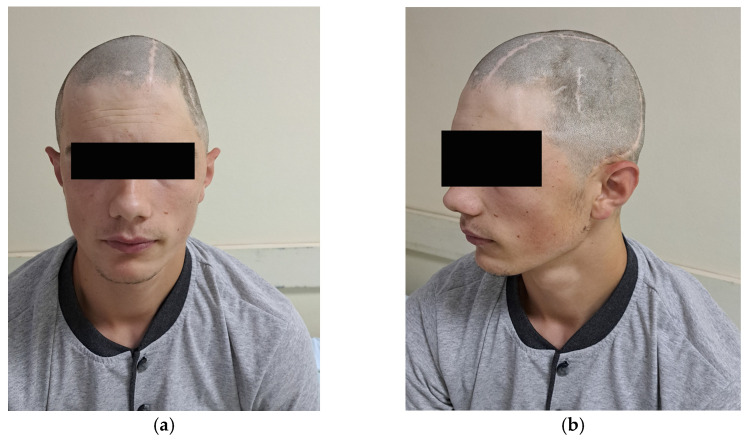
Second case with (**a**) frontal and (**b**) lateral aspect preoperative images.

The second case describes a 19-year-old male patient who experienced a traumatic brain injury (TBI) from a fall at a height of 7 m. Prior to the CP surgery, the patient had undergone a previous operation for the same injury at a different medical facility three years earlier. The cranial defect involved the frontal, parietal, and temporal bones. The duration between injury and CP was 36 months, and the surgical operation lasted 120 min (Figure 5).

The third case involves a 49-year-old female patient who underwent DC surgery due to a Middle Cerebral Artery (MCA) aneurysm, resulting in left-sided hemiparesis and paresis of the right oculomotor and facial nerve. Three years before the CP procedure, the patient had previously undergone treatment at a different medical facility in another state. The cranial defect involved the frontal and temporal bones. The time interval between the injury and CP was 36 months, and the surgical operation lasted 145 min.

According to the classification system proposed by Poukens et al. [15] case 1 and 3 were categorized as grade II, while case 2 was graded III. This classification signifies that the defects observed are greater than 5 cm^2^ but are equal to or smaller than 100 cm^2^ in size. The table includes the respective defect sizes in millimeters for each case, which are 119.8 mm × 52.8 mm for Case 1 (63.3 cm^2^), 121.0 mm × 89.5 mm for Case 2 (108.3 cm^2^), and 102.2 mm × 68.1 mm for Case 3 (69.6 cm^2^) (Table 1).

### 3.2. Radioogical Findings

**Figure 6:**
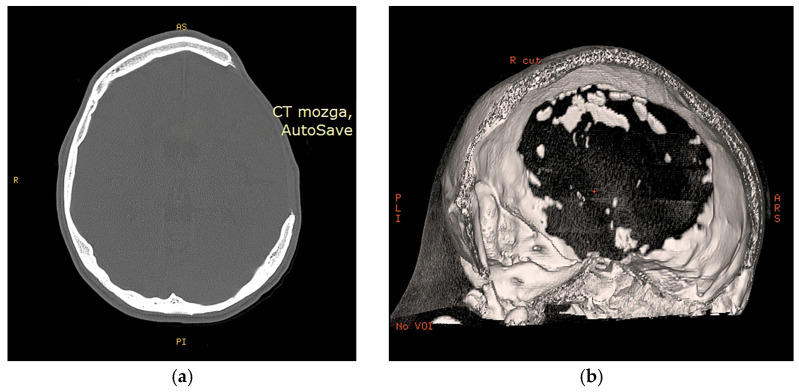
Second case CT scan: (**a**) transverse slice, and (**b**) CT-based 3D reconstruction.

**Figure 7:**
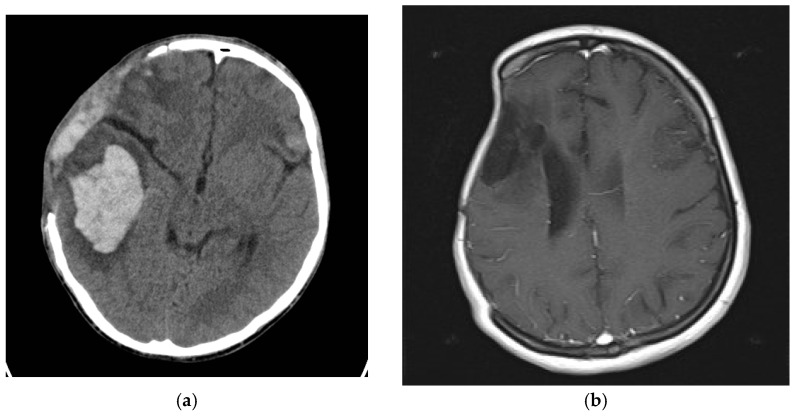
Presence of intracerebral bleeding in first case (**a**) after DC ischemic zone in the right frontal, and parietal lobes due to MCA occlusion in third case (**b**).

In Figure 6a, a defect affecting the frontal, temporal, and parietal bones of the skull vault can be observed in case 2. Based on the CT scan, a 3D reconstruction was made, which is shown in Figure 6b for better visualization and surgical planning. In the case of Case 1, intracerebral bleeding is visible as a result of TBI, as well as the state after DC (Figure 7a). As can be seen in Figure 7b, Case 3 has an ischemic zone in the right frontal and parietal lobes due to MCA occlusion, as well as a large bone defect.

### 3.3. Intraoperative Processing

**Figure 8:**
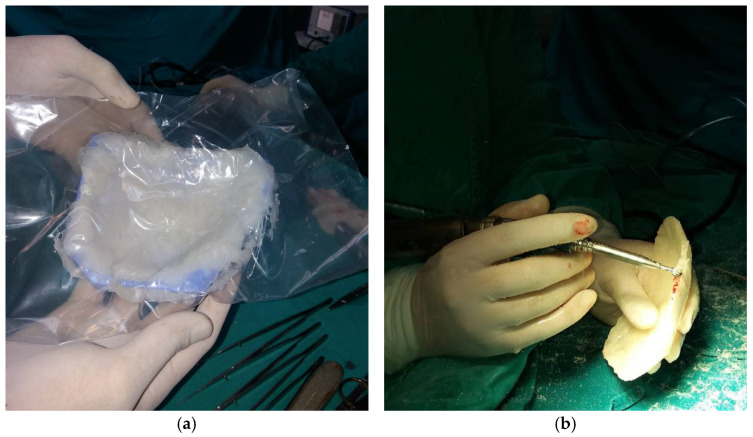
Intraoperative processing of the PMMA implant: (**a**) PMMA mass adapting using mold; (**b**) grinding and finishing of implant.

**Figure 9:**
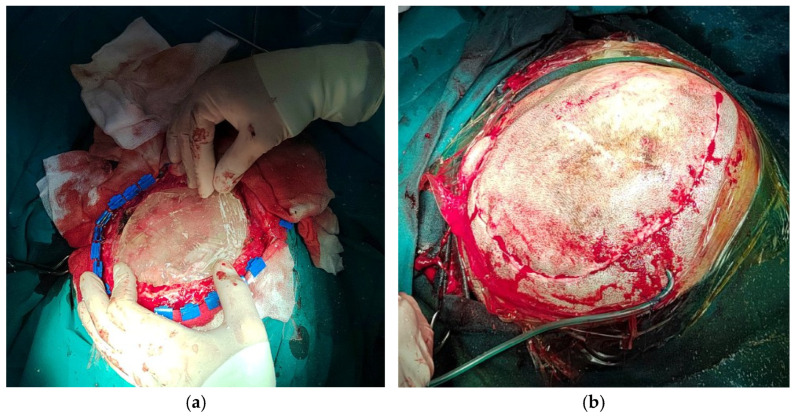
Implantation of PMMA implant (**a**), and closure (**b**).

During the intraoperative processing of the implant, the PMMA mass was adapted using a mold (Figure 8a), and subsequently subjected to grinding and finishing procedures (Figure 8b) to achieve optimal congruence between the implant and the bone defect. In Case 2, dry sterilization resulted in the mold fracturing, necessitating the creation of a new mold based on the 3D prototype, followed by adaptation. Following these steps, implantation was performed (Figure 9a), and the scalp was closed (Figure 9b).

### 3.4. Technological Process and Costs

In Cases 1 and 2, the material costs were USD 99 and USD 90, respectively, while Case 3 incurred the same material cost as Case 2, totaling USD 90. Remarkably, the technical processing costs for all three cases were consistent, with a value of USD 395 for Cases 1 and 2, and USD 400 for Case 3. As a result, the overall expenses, including both material and processing costs, for Cases 1, 2, and 3 amounted to USD 494, USD 485, and USD 490, respectively. It is noteworthy that the technical processing costs were voluntarily undertaken by the author (D.S.) in collaboration with the Faculty of Mechanical Engineering, University of Zenica (Table 2).

### 3.5. Outcome and Aestetic Satisfaction

**Figure 10:**
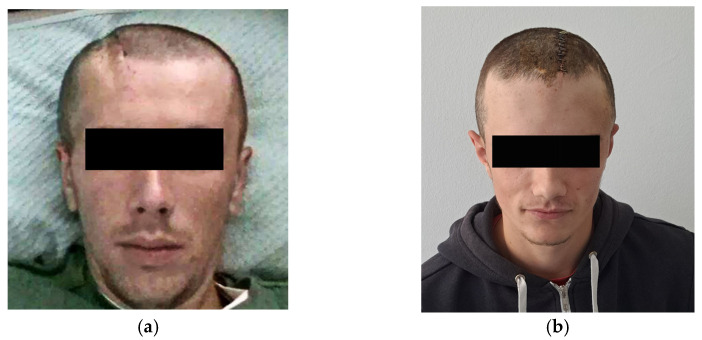
Presentation of Case 1 (**a**) after the surgical procedure, after the removal of the stapler pins, after 10 days, and Case 2 (**b**) postoperatively 6 days before the removal of the stapler pins.

During the follow-up, all cases showed significant aesthetic progress along with functional improvements. However, Case 3, due to prior neurological deficits, experienced unchanged conditions. The first two cases exhibited no visible neurological impairments. Figure 10a illustrates the aesthetic enhancements achieved in Case 1 after the CP procedure, while Figure 10b corresponds to Case 2, demonstrating similar aesthetic improvements following the CP.

**Figure 11:**
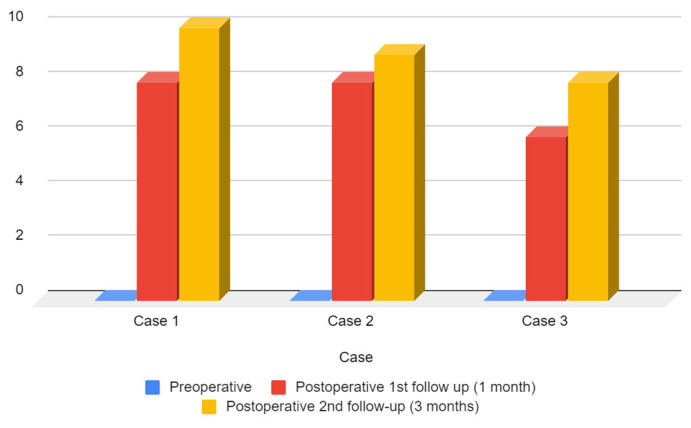
Preoperative and follow-up ANA score.

One month post-surgery, the ANA scores for Cases 1 and 2 increased to 8, indicating improved aesthetic satisfaction. Case 3 also demonstrated improvement, albeit to a lesser extent, with an ANA score of 6 (Figure 11). At the three-month follow-up, aesthetic outcomes continued to progress. Both Case 1 and Case 2 achieved higher ANA scores of 9 and 10, respectively, indicating further enhancement in their aesthetic appearance. Similarly, Case 3 exhibited continued improvement, attaining an ANA score of 8.

After the surgical procedure, in all three cases, both functional and aesthetic effects were achieved. In Figure 10a, Case 1 can be seen after the removal of the stapler pins, showing evident aesthetic improvement. In Figure 10b, Case 2 can be observed with aesthetic and functional progress after the cranionoplastic neurosurgical procedure.

## 4. Discussion

CP is a complex surgical procedure aimed at reconstructing cranial defects [17]. Bosnia and Herzegovina, being a middle-income country, faces healthcare challenges due to resource constraints [18,19,20]. This encompasses limitations in funding, infrastructure, and access to advanced medical technologies [21,22]. Innovative approaches are imperative for optimizing healthcare delivery [22,23,24,25,26]. Despite these hurdles, this study demonstrates the successful implementation of CP within limited resources.

Three cases with positive outcomes in functional and aesthetic contexts were presented earlier. The first two cases were male, aligning with the incidence of TBI and the 2:1 male-to-female ratio according to Maegele et al. [27]. According to Aydin et al. [1], TBI is the most common cause of DC followed by CP. In the third case, the cause was a surgical procedure involving an aneurysm of the middle cerebral artery that necessitated DC, a common occurrence according to Zijlstra et al. [28].

The size of the cranial defect varied. The largest defect was observed in Case 2, measuring 108.3 cm^3^ (Figure 6), categorizing it as grade III according to Poukens et al. [15]. The size of the cranial defect is of paramount importance as it dictates the complexity of the surgical procedure. Typically, DC requires removal of a bone flap ranging from 5–100 cm^2^, corresponding to Poukens et al.’s [15] grade II. It is less common for this procedure to necessitate DC exceeding 100 cm^3^. Moreover, Johnson et al. [29] state that a craniotomy larger than 125 cm^2^ poses a risk of flap failure.

The time interval from injury or DC in our cases ranged from 12 to 36 months. The average duration from DC to CP according to the study by Quah et al. [30] was 23 months. Prolonging CP is associated with the development of hydrocephalus as per Morton et al. [31]. Additionally, prolongation carries the risk of infection and prolonged antibiotic use [30], while timely CP within 3 months of DC reduces neurological complications, especially in TBI patients [32]. Therefore, timely intervention within 3 months is a key factor in improving patient outcomes and reducing costs in LIMCs. The length of hospitalization was notable in Case 3 (31 days) due to accompanying conditions, emphasizing the need to consider existing comorbidities when initiating a CP procedure.

Ultimately, the use of PMMA, its accessibility [33], safety, and reliability for medical purposes [34], as well as its ease of manual manipulation [35], constitute crucial considerations in LIMCs. Its applicability in CPs, even in restrictive conditions, represents an acceptable modality for addressing cranial defects. The effectiveness in limited conditions was affirmed by a randomized controlled trial by Mansouri et al. [36], where this material was used as an implant in deep sclerectomy. Furthermore, Ashraf et al. [37] offered significant insights in their Pakistani study regarding the application of 3D-printing-mediated PMMA in CP, stating that its utilization holds potential clinical, aesthetic, and radiological benefits in LIMIC conditions.

### Overview of Implemented (Bosnian) Model for CP in LIMCs

**Figure 12:**
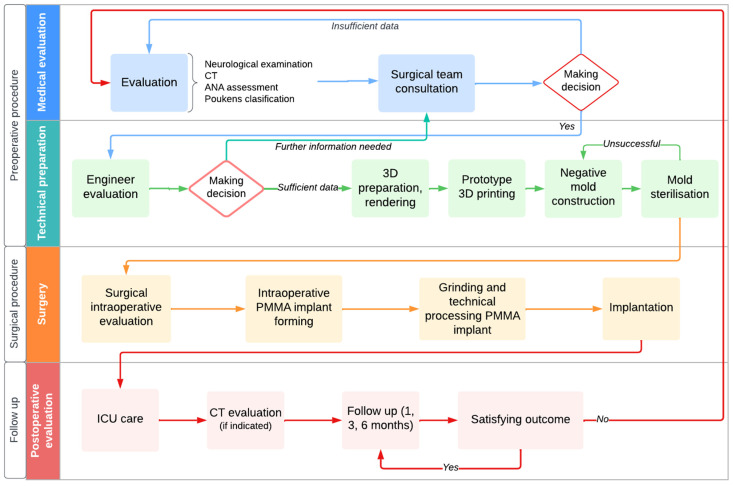
Overview chart with proposed steps for implementing the process and procedure of CP in LIMCs.

Preoperative 3D CP involves medical assessment and technical processing (Figure 12). Medical evaluation includes defect examination, medical history, and health status [13,27]. CT scans are cost-effective and efficient for evaluation [28]. Advanced CT technology provides clear visualization for precise measurement [31]. Reconstruction enhances preoperative understanding [38,39,40]. Freely available software meets clinical standards [41,42,43,44], ensuring widespread accessibility.

After medical evaluation, 3D software (e.g., MIMICS, version 10.01) processes CT data for precise skull reconstruction [45,46,47,48,49]. Cutting and mirroring techniques virtually close the defect, ensuring an accurate fit for the prosthesis. This phase is crucial for precise implant customization [14,48]. The 3D CP team comprises neurosurgeons and engineers [50,51]. Neurosurgeons handle patient assessment, surgery, and treatment decisions. Engineers use specialized software for precise 3D modeling of the cranial defect and customizing the prosthesis [52,53]. This collaboration enhances the accuracy and effectiveness of the procedure [43,44]. In our approach, an external engineer provided technical consultation, optimizing 3D CP. Their expertise in imaging and modeling led to tailored prostheses, reducing costs and increasing accessibility in resource-limited systems [54,55]. Neurosurgeon-led evaluation, alongside preoperative classification, initiates CP. Considering both functionality and aesthetics is vital. Maricevich et al. [56] emphasize aesthetic satisfaction’s impact on patient well-being. Enhancing both aspects aids reintegration and boosts confidence [57,58], enhancing overall quality of life.

PMMA, hydroxyapatite, and titanium are commonly used materials in medical applications, each with distinct properties and risks [59]. PMMA and hydroxyapatite are non-conductive and radiolucent, suitable for imaging. PMMA’s carcinogenicity is debated, with studies showing no clear cancer-causing effects [60]. PMMA is FDA-approved for various medical devices [51] and widely used in European countries [33,61,62,63,64]. PMMA offers good mechanical resistance, while hydroxyapatite excels in osteointegration [57,58,59]. Hydroxyapatite has a higher infection rate [59,60,61]. Titanium is strong but not suitable for pediatric use and is the most expensive material [12]. PMMA is cost-effective and radiolucent, allowing various imaging methods. PMMA’s strength, stability, and cost-effectiveness make it preferred for manufacturing prostheses. It may cause immunologic reactions but can be combined with antibiotics to reduce the risk of infection [64,65,66,67,68,69,70,71,72,73]. In 3D CP, PMMA yields favorable cosmetic results with low complications [64,65,66,67,68,69,70,71,72,73]. PMMA is the most cost-effective option for CP in LIMCs [65,66,67,68,69,70,71,72,73,74].

After preoperative steps and multidisciplinary collaboration, the surgical intervention begins (Figure 12). The procedure involves examining the defect, and modifying the PMMA mass with a sterilized mold [14]. Two approaches for PMMA implantation exist: preoperative fabrication (costlier and riskier) and intraoperative modification (more suitable for LIMCs, potentially lengthening surgery) [12,75]. The average surgical duration for the presented cases was 135 min [76,77]. Following modification, the PMMA implant is placed over the bone defect and secured with silk sutures [78,79,80]. Postoperative monitoring involves ICU care and neurosurgical oversight. CT scans are used if complications are suspected. Our cases had varying hospital stays, with no complications reported. Follow-ups are scheduled at one, three, and six months, including neurological and physical assessments, and aesthetic evaluation with the ANA scale.

## 5. Conclusions

In conclusion, our Bosnia and Herzegovina model, employing a multidisciplinary team and advanced 3D-based technologies, offers a cost-effective approach for cranial defect management in LIMCs. The use of PMMA, with intraoperative modification, enhances cost-effectiveness and reduces complications. Successful outcomes underscore the value of collaboration between medical and engineering professionals in LIMCs. Further research with larger cohorts, long-term follow-ups, and cost-effectiveness analyses for different implant materials are warranted. Advances in 3D imaging and printing hold promise for refining craniofacial reconstruction in resource-constrained settings. This model provides a valuable framework for LIMCs to adopt innovative approaches, ultimately enhancing outcomes and quality of life for craniofacial reconstruction patients.

## Figures and Tables

**Table 1:** Baseline characteristics of patients.

Case	1	2	3
Age	19	24	49
Years	Male	Male	Female
Cause	SDH, TBI caused by falling from a height of 7 m	TBI caused by falling from the roof of a house from a height of 4–5 m	Cranial decompression due to surgery of ACM aneurysm
Anamnestic data	Treated surgically 3 years b.c. in another facility due to previously explained injury	Treated surgically two years ago in our facility, epidural empyema without isolating the pathogen a year ago	Treated three years b.c. in another facility (and state) due to MCA aneurysm, present hemiparesis of the left side, as well as paresis of the right oculomotor and facial nerve
Defect size	119.8 mm × 52.8 mm (63.3 cm^2^)	121.0 mm × 89.5 mm (108.3 cm^2^)	102.2 mm × 68.1 mm (69.6 cm^2^)
Side	Left	Right	Left
Localisation	Frontal, parietal and temporal bone included	Frontal, parietal and temporal bone included	Frontal and temporal bone included
Poukens et al. [15] classification	II	III	II
Time to CP (months)	36	12	36
Operation time	120	140	145
Hospitalisation days	5	11	31
Comorbidities	None	None	Status after MCA occlusion
Outcome	Functional and aesthetic improvement	Functional and aesthetic improvement	Functional and aesthetic improvement

b.c.—before CP.

**Table 2:** Costs of material and technical processing.

Case	1	2	3
Material costs	USD 99	USD 90	USD 90
Technical processing costs ^1^	USD 395	USD 395	USD 400
Total costs material and processing costs	USD 494	USD 485	USD 490

^1^ The technical processing costs represent an approximation of expenses incurred during the technical processing phase. The technical processing was carried out on a voluntary basis by the author (D.S.) in collaboration with the Faculty of Mechanical Engineering, University of Zenica.

## Data Availability

The data used to support the findings of this study are available from the corresponding author upon request.

## Supplementary Materials

The following supporting information can be downloaded at: https://www.mdpi.com/article/10.3390/medicina59101732/s1, Video S1: The 3D modeling technique based on Fused Filament Fabrication (FFF).
